# Indirect Adventitious Shoot Organogenesis and Plant Regeneration from *Solanum nigrum* L.

**DOI:** 10.3390/plants15203115

**Published:** 2026-10-12

**Authors:** Jingjing Zhang, Qi Guo, Xinyu Cui, Tiantian Sun, Zihan Fan, Xinting Ma, Jinhui Gao, Jingli Yang, Shicheng Zhao

**Affiliations:** 1 School of Pharmacy, Harbin University of Commerce, Harbin 150076, China; m15192876183@163.com; 2 Institute of Forestry Science of Heilongjiang Province, Harbin 150081, China; guoqinefu@163.com; 3 State Key Laboratory of Tree Genetics and Breeding, Northeast Forestry University, Harbin 150040, China; cxy17852849591@163.com (X.C.); sun025763@163.com (T.S.); fanshi26528@163.com (Z.F.); matt019@163.com (X.M.); yifan85831647@163.com (J.Y.); 4 Yichun Branch of Heilongjiang Academy of Forestry, Yichun 153000, China; zhl737603@163.com

**Keywords:** *Solanum nigrum* L., plant growth regulators, adventitious shoots, regeneration

## Abstract

*Solanum nigrum* L. plays an important role in anti-tumor activity. Previous studies have reported an imperfect in vitro regeneration system with unclear regeneration processes. This study firstly screened the optimal explant type for regeneration, and leaf explants were finally selected as the experimental materials. On this basis, the effects of plant growth regulator combinations and sucrose concentrations on adventitious shoot induction, shoot elongation and rooting were systematically investigated using leaf explants. The results showed that leaf explants exhibited the highest adventitious shoot induction capacity, with an induction rate of 100.00%, significantly higher than petioles (71.33%) and stem segments (51.67%). Using leaf explants, the optimal Murashige and Skoog (MS) medium for adventitious shoot induction contained 2.0 mg·L^−1^ 6-benzyladenine (BA), 0.1 mg·L^−1^ α-naphthaleneacetic acid (NAA), 30 g·L^−1^ sucrose and 0.7% (*w*/*v*) agar, achieving a stable adventitious shoot induction rate of 100.00%. The optimal medium for adventitious shoot elongation was MS supplemented with 0.5 mg·L^−1^ 6-BA, 0.1 mg·L^−1^ NAA, 30 g·L^−1^ sucrose and 0.7% (*w*/*v*) agar, with an average of 27.67 elongated adventitious shoots per explant. The optimal rooting medium was half-strength MS medium containing 0.1 mg·L^−1^ NAA, 30 g·L^−1^ sucrose and 0.7% (*w*/*v*) agar, with a rooting rate of 96.67%. This study successfully established a complete and efficient tissue culture system for *S. nigrum*, which will facilitate large-scale propagation of this medicinally valuable plant.

## 1. Introduction

*Solanum nigrum* L. is an annual herbaceous plant of the family Solanaceae. It is widely distributed in most regions of China, especially in the Yangtze River Basin and southern hilly areas. *S. nigrum* mainly contains steroidal alkaloids, steroidal saponins, flavonoids, lignans and other components, exerting anti-inflammatory, anti-tumor, anti-oxidant and other pharmacological effects [1,2]. Pharmacological studies have shown that the anti-tumor activity of *S. nigrum* is mainly attributed to solanine, solamargine, and solasonine [3,4,5]. Currently, the cultivation of *S. nigrum* mainly relies on seed germination to form complete plants. However, its seeds are small and exhibit problems such as inconsistent germination time, uneven seedling emergence, and difficulty in maintaining superior traits [6].

Plant tissue culture features multiple advantages including a high propagation coefficient, short growth cycle, preservation of desirable plant traits, and freedom from seasonal constraints [7]. As a commonly used medicinal plant, *S. nigrum* has been investigated in previous studies. Considerable variations exist in culture conditions adopted by different researchers, such as explant types, medium formulations, and plant growth regulators (PGRs). For instance, Subba et al. [8] and Kavitha et al. [9] used shoot tips as explants to induce adventitious shoots; Younes et al. [10] employed stem segments for rapid propagation of *S. nigrum*; and Geetha et al. [11] used leaf explants with supplementary silver nitrate to achieve adventitious shoot induction. Although several reports are available on the tissue culture of *S. nigrum*, most existing studies focus only on individual regeneration stages. Few investigations have systematically explored the key techniques covering the full pipeline, including explant screening, adventitious shoot induction, elongation, rooting and acclimatization. Furthermore, the origin of adventitious shoots remains unclear.

Therefore, in this study, different explant types were firstly screened to select the optimal explant source for *S. nigrum* regeneration. After comparative evaluation, leaf explants were confirmed as the most suitable material and were subsequently used to systematically screen plant growth regulator combinations and sucrose concentrations for each stage of the tissue culture workflow. The optimal culture conditions for adventitious shoot induction, shoot elongation and rooting were determined to establish a stable and efficient tissue culture propagation system for *S. nigrum*. This work provides technical support for germplasm conservation, rapid propagation of high-quality seedlings and industrial breeding of this medicinal species.

## 2. Results

### 2.1. Effect of Explants on Adventitious Shoot Induction

**Figure 1:**
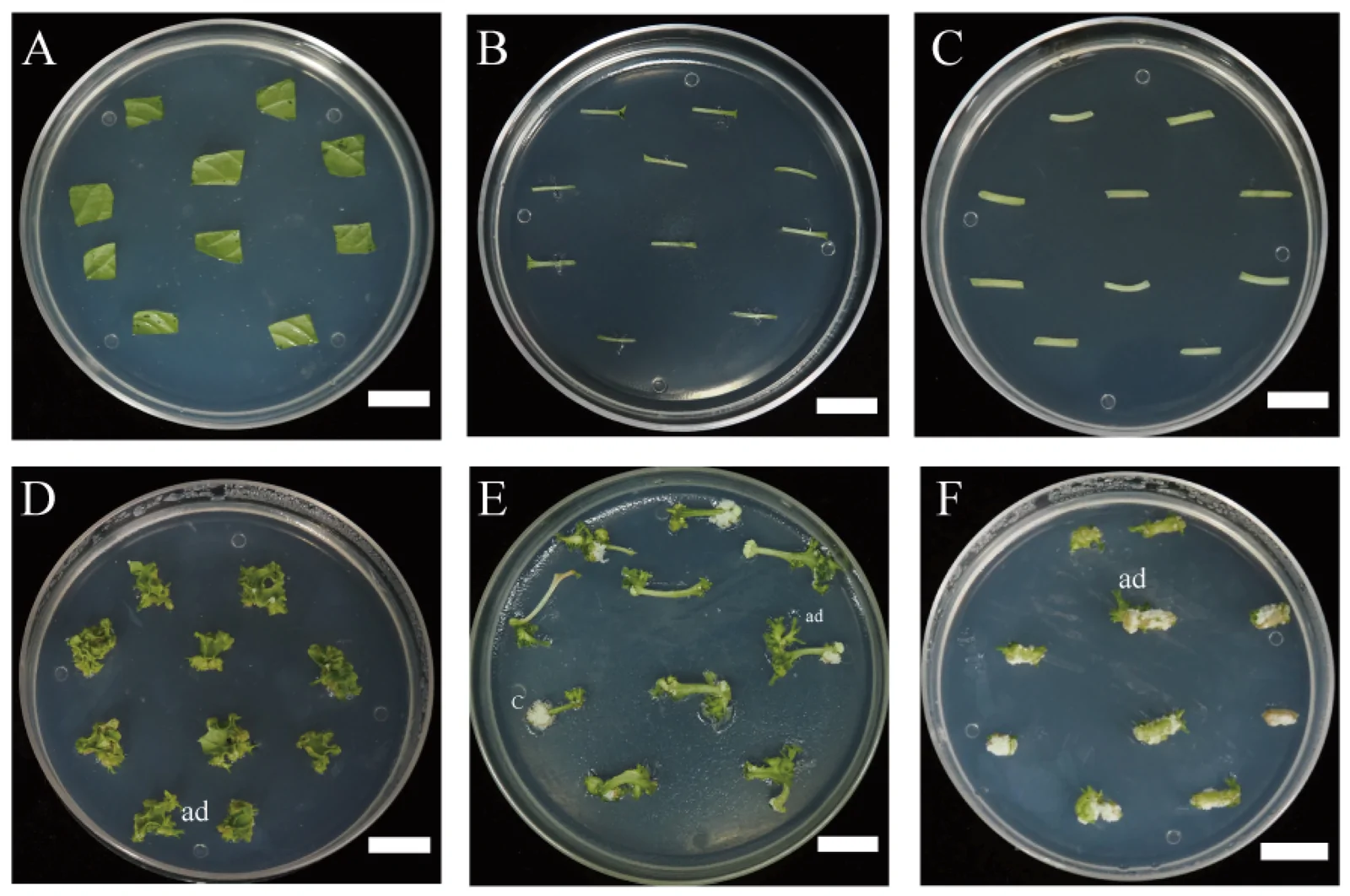
The effect of explants on adventitious shoot induction. (**A**) Leaves of *S. nigrum*; (**B**) petioles of *Solanum nigrum* L.; (**C**) stem segments of *S. nigrum*; (**D**) the morphology of adventitious shoots induced from *S. nigrum* leaves on the 20th day; (**E**) the morphology of adventitious shoots induced from *S. nigrum* petioles on the 20th day; (**F**) the morphology of adventitious shoots induced from *S. nigrum* stem segments on the 20th day. Bar = 1 cm. Adventitious shoots = ad; calluses = c.

Explant type significantly affected differentiation capacity, directly altering the efficiency of adventitious shoot induction. Leaves, petioles, and stem segments of *S. nigrum* served as explants (Table 1). The results showed that adventitious shoots could be induced from all types of explants. Leaf explants exhibited the highest adventitious shoot number (9.87) and induction rate (100.00%), followed by petiole explants with an adventitious shoot number of 2.20 and an induction rate of 71.33%. Stem segments produced the lowest values, with an adventitious shoot number of 2.13 and an induction rate of 51.67% (Table 1). After placing the explants on the culture medium and approximately 6–8 days of light culture, leaf blades curled, and loose white calluses formed at the cut ends of stem segments, and petioles began to swell. On the 12th day, green shoot primordia appeared at the leaf margins, and the cut ends of stem segments accompanied by the formation of loose white calluses on stem segments. Adventitious shoot differentiation from petiole explants was slower, with green shoot primordia mainly distributed at both ends of the petioles. On the 20th day, the shoot primordia gradually developed into adventitious shoots and continued to elongate gradually with time. Adventitious shoots induced from leaf blades displayed a vibrant green color and vigorous growth (Figure 1D). Those induced from stem segments were sparse, thick, and short, often accompanied by white friable calluses at both ends (Figure 1F). Although adventitious shoot differentiation from petioles was slower compared to other explants, the induced shoots were emerald green, slender, and more numerous than those from stem segments (Figure 1E).

### 2.2. Effect of Basal Media on Adventitious Shoot Induction

**Figure 2:**
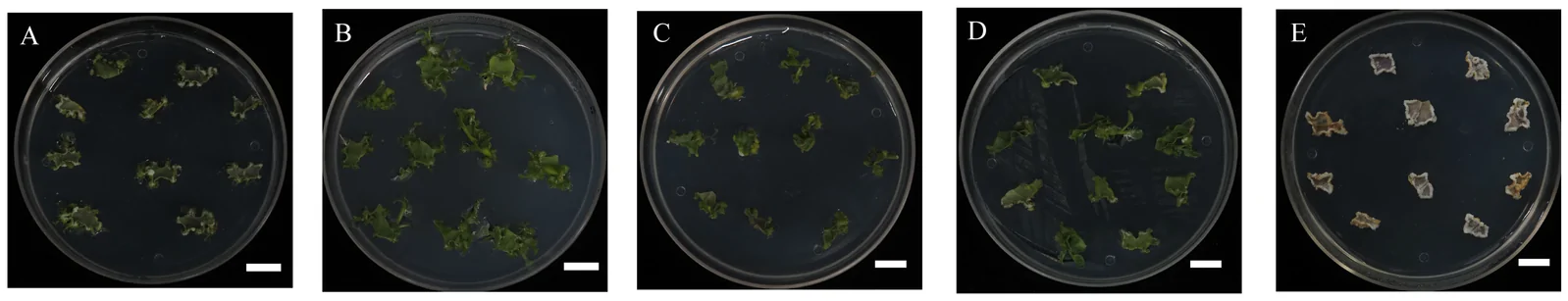
Effects of different media concentrations on adventitious shoot induction. (**A**) Leaf explants cultured on 1/2 MS medium; (**B**) leaf explants cultured on MS medium; (**C**) leaf explants cultured on B5 medium; (**D**) leaf explants cultured on N6 medium; (**E**) leaf explants cultured on White medium; Bar = 1 cm.

Five basal media including 1/2 MS, MS, B5, N6, and White were employed to induce adventitious shoot formation using leaf explants only, and the induction rates were in the order of MS > 1/2 MS > B5 > N6 > White. Among these, the MS medium achieved the highest adventitious shoot induction rate of 100.00%, yielding an average of 8.40 adventitious shoots per explant. The adventitious shoot induction rates on 1/2 MS, B5, and N6 media were all lower than those on MS medium, at 89.33%, 87.67%, and 76.67%, respectively (Table 2). The adventitious shoots exhibited robust growth, presenting a healthy, light green color and strong vitality (Figure 2B). On 1/2 MS, B5, and N6 media, some adventitious shoots produced white, loose calluses, and the inoculated leaves appeared darker in color with weaker vitality compared to those on MS medium (Figure 2). In contrast, White medium failed to induce any adventitious shoots, and the inoculated leaves became dry, wilted, and yellowed (Figure 2E). Consequently, MS medium is identified as the most suitable medium for adventitious shoot induction in *S. nigrum*.

### 2.3. Effect of Plant Growth Regulators (PGRs) on Adventitious Shoot Induction

Levene’s test verified homogeneity of variances (*F* = 1.675, *p* = 0.108). Two-way analysis of variance (ANOVA) revealed that 6-BA concentration, NAA concentration, and their interaction all exerted highly significant effects on adventitious shoot induction number (*p* < 0.001). 6-BA exhibited a stronger independent regulatory effect, and there was an interactive effect between the two plant PGRs (Table 3).

**Figure 3:**
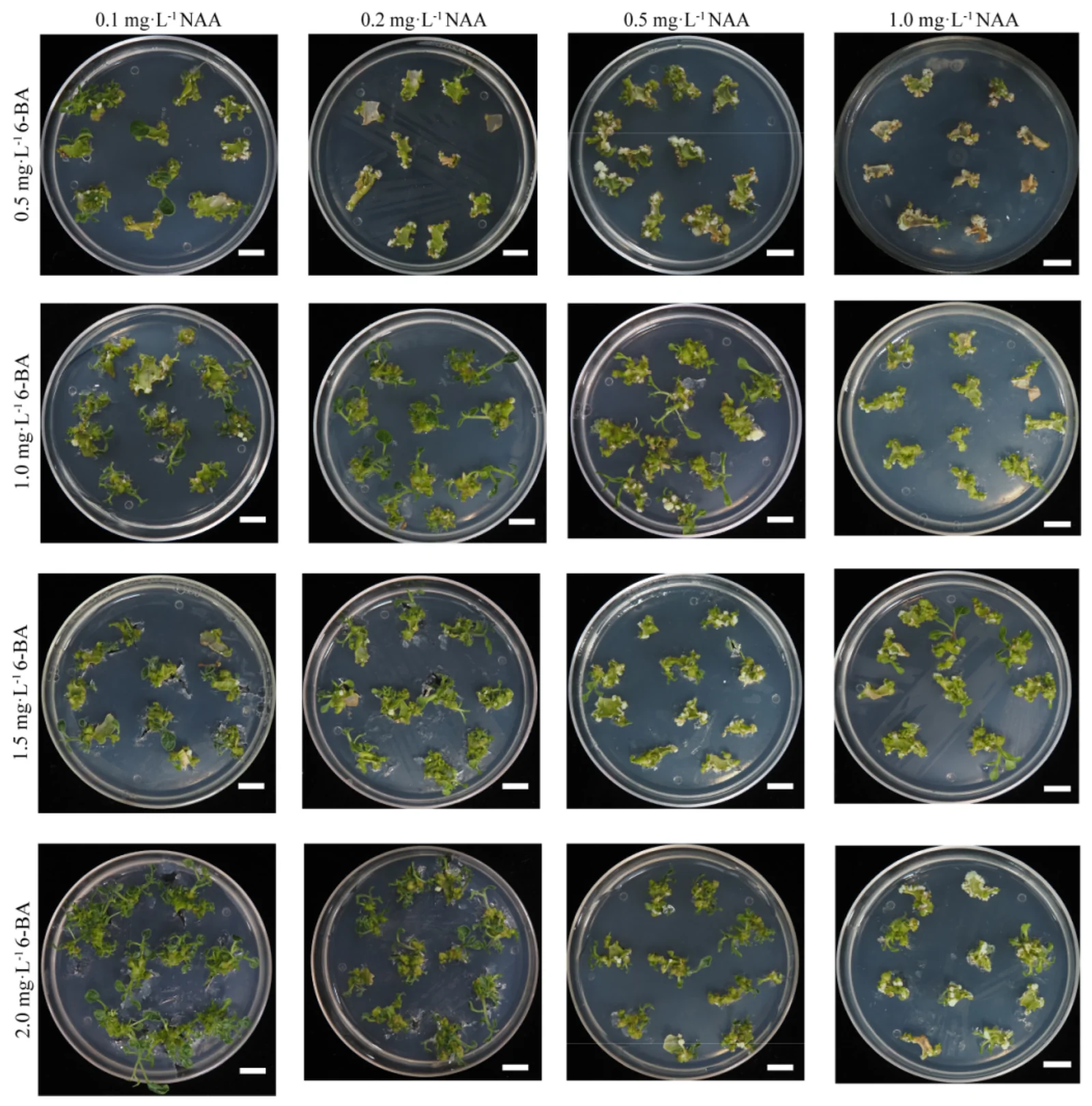
Morphological phenotypes of *S. nigrum* explants under different plant growth regulator combinations. Images display callus formation, browning, and adventitious shoot regeneration performance of explants cultured on media with 16 combinations of NAA and 6-BA. Corresponding quantitative data are summarized in Table 3. From left to right: 0.1, 0.2, 0.5 and 1.0 mg·L^−1^ NAA. From top to bottom: 0.5, 1.0, 1.5 and 2.0 mg·L^−1^ 6-BA. Bar = 1 cm.

Within the concentration range tested, the average number of adventitious shoots and shoot induction rate generally increased with rising 6-BA concentration. When the 6-BA concentration was kept constant, the average number of adventitious shoots decreased overall as NAA concentration increased. Meanwhile, inoculated leaf explants exhibited drying and yellowing symptoms. Among all treatment combinations, the combination of 2.0 mg·L^−1^ 6-BA and 0.1 mg·L^−1^ NAA exhibited the best performance, yielding an average of 8.97 adventitious shoots and a shoot induction rate of 100.00%, which was significantly higher than that obtained with the other PGR combinations (*p* < 0.05) (Table 4). These adventitious shoots exhibited elongated leaf morphology and robust growth (Figure 3).

### 2.4. Effect of Sucrose on Adventitious Shoot Induction

**Figure 4:**
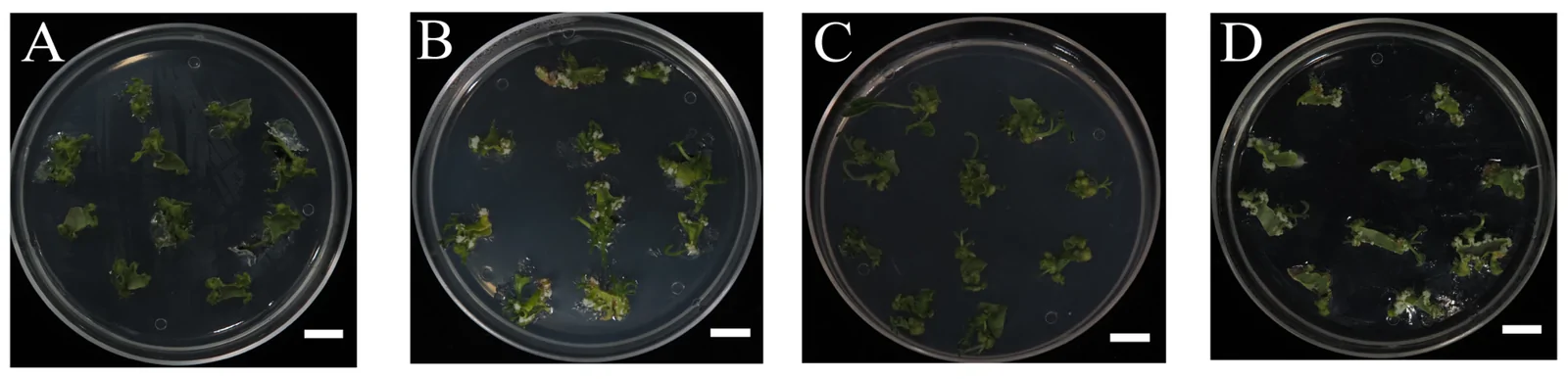
Effects of different sucrose concentrations on adventitious shoot induction. (**A**) Leaf explants cultured on MS medium with 10 g·L^−1^ sucrose; (**B**) leaf explants cultured on MS medium with 20 g· L^−1^ sucrose; (**C**) leaf explants cultured on MS medium with 30 g·L^−1^ sucrose; (**D**) leaf explants cultured on MS medium with 40 g·L^−1^ sucrose; Bar = 1 cm.

The results showed that when 30 g·L^−1^ sucrose was added to the medium, the adventitious shoot induction rate was the highest (100.00%), yielding an average of 8.87 adventitious shoots (Table 5). The induced adventitious shoots had longer stems and produced almost no white calluses. Under treatment with 10 or 20 g·L^−1^ sucrose, the leaves remained emerald green, but the adventitious shoots were relatively tender, fragile, and accompanied by a small amount of loose white calluses. In contrast, under treatment with 40 g·L^−1^ sucrose, some adventitious shoots exhibited light purple leaves due to excessive osmotic pressure, with more loose white calluses appearing at the leaf margins, and the adventitious shoots were slender and weak (Figure 4). Therefore, 30 g·L^−1^ sucrose was considered the optimal carbon source for adventitious shoot induction in *S. nigrum*.

### 2.5. Elongation of Adventitious Shoots

Shoot clusters with tender leaves and approximately 1 cm long stems were inoculated into elongation medium. The results showed that the combination of 0.5 mg·L^−1^ 6-BA and 0.1 mg·L^−1^ NAA resulted in a higher number of elongated adventitious shoots, with thicker stems and darker plant coloration. As the NAA concentration gradually increased, the number of elongated adventitious shoots progressively decreased, and the shoots became stunted, with smaller or even withered leaves (Table 6). Therefore, the optimal medium for adventitious shoot elongation in *S. nigrum* was MS medium supplemented with 0.7% (*w*/*v*) agar, 30 g·L^−1^ sucrose, 0.5 mg·L^−1^ 6-BA and 0.1 mg·L^−1^ NAA.

### 2.6. Rooting

**Figure 5:**
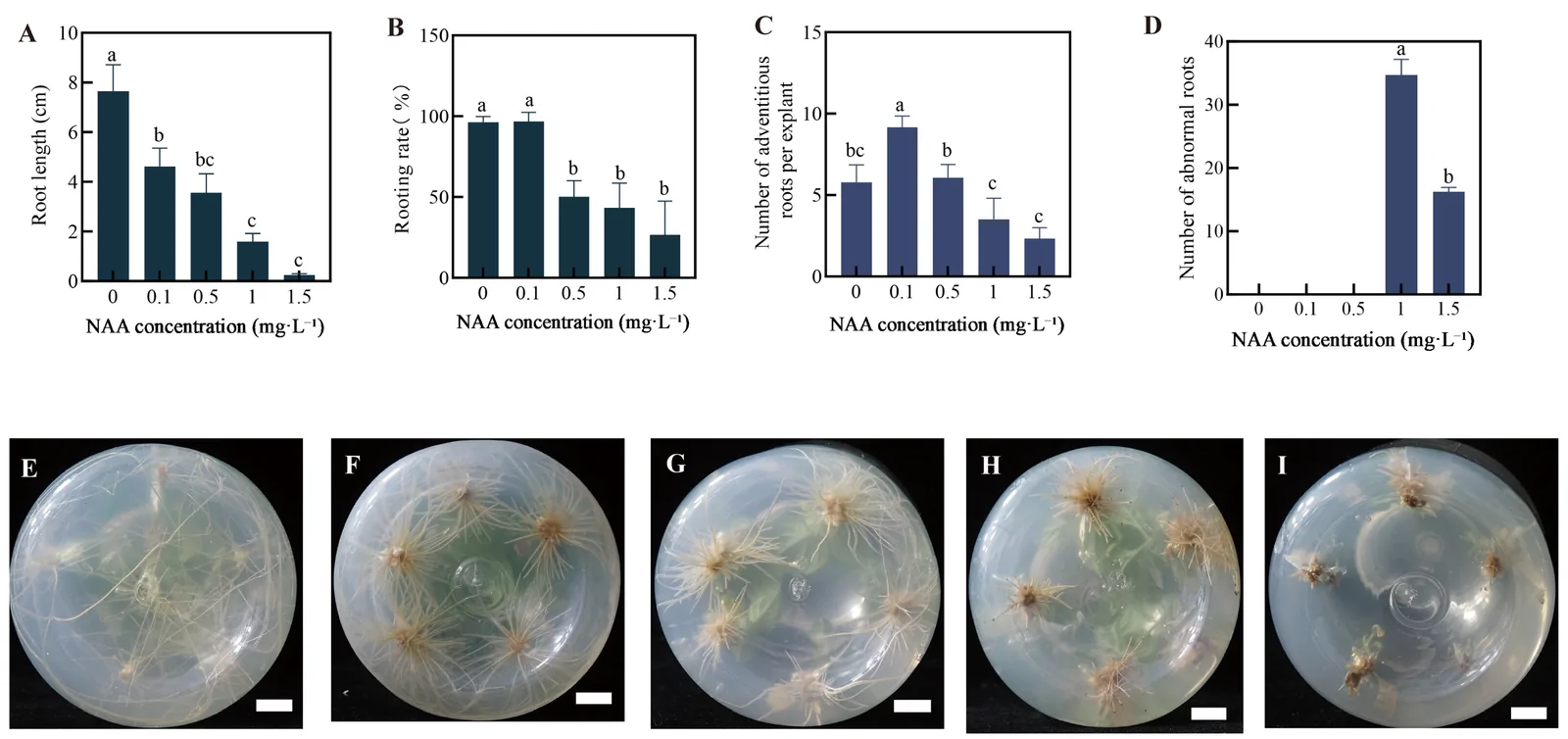
Rooting. (**A**) 1/2 MS medium without NAA; (**B**) 1/2 MS medium was supplemented with 0.1 mg·L^−1^ NAA; (**C**) 1/2 MS medium was supplemented with 0.5 mg·L^−1^ NAA; (**D**) 1/2 MS medium was supplemented with 1.0 mg·L^−1^ NAA; (**E**) 1/2 MS medium was supplemented with 1.5 mg·L^−1^ NAA; (**F**) average root length at varying NAA concentrations; (**G**) count of abnormal adventitious roots under different NAA concentrations; (**H**) root induction rate with different NAA concentrations; (**I**) total number of adventitious roots per explant at various NAA concentrations. Bar = 1 cm. Different lowercase letters within the same column indicate significant differences among treatments at *p* < 0.05.

Quantitative statistical analysis revealed that the average root length decreased significantly with increasing NAA concentration. The control group (0 mg·L^−1^ NAA) showed a markedly longer average root length than all NAA-treated groups, followed by the 0.1 mg·L^−1^ NAA treatment (Figure 5A). No significant difference in rooting induction rate was detected between the 0 and 0.1 mg·L^−1^ NAA groups, whereas rooting rates dropped sharply once the NAA concentration exceeded 0.5 mg·L^−1^ (Figure 5B). The average number of adventitious roots per explant was significantly affected by NAA concentration. The highest average root number (9.16 ± 0.68) was obtained at 0.1 mg·L^−1^ NAA. As NAA concentration increased, the average number of adventitious roots per explant gradually decreased. The 1.0 and 1.5 mg·L^−1^ NAA treatments produced the lowest average adventitious root numbers (Figure 5C). Numerous abnormal roots were observed under 1.0 and 1.5 mg·L^−1^ NAA treatments (Figure 5D). Comprehensive evaluation of rooting rate, total adventitious root number, root morphology and root length identified 0.1 mg·L^−1^ NAA as the optimal concentration for root induction.

Well-grown adventitious shoots were excised and inoculated onto rooting medium. For the medium without NAA, the roots derived from adventitious shoots were thin and slender with the maximum elongation length, yet they grew irregularly, accompanied by delayed initial rooting and low root formation (Figure 5E). As the NAA concentration increased gradually, roots became thickened with enhanced elongation, growing in an orderly pattern around the basal part of explants, while leaf expansion was also observed (Figure 5). At an NAA concentration of 0.1 mg·L^−1^, adventitious shoots differentiated a large number of healthy, uniformly distributed root systems; the rooting rate peaked at 96.67%, and regenerated plantlets exhibited robust growth (Figure 5F). When the NAA concentration was further elevated to 0.5–1.5 mg·L^−1^, the number of differentiated roots decreased continuously, root length was shortened, and roots turned yellow (Figure 5G and Figure 5H); massive abnormal roots emerged under high NAA concentrations (Figure 5I).

### 2.7. Transplantation

**Figure 6:**
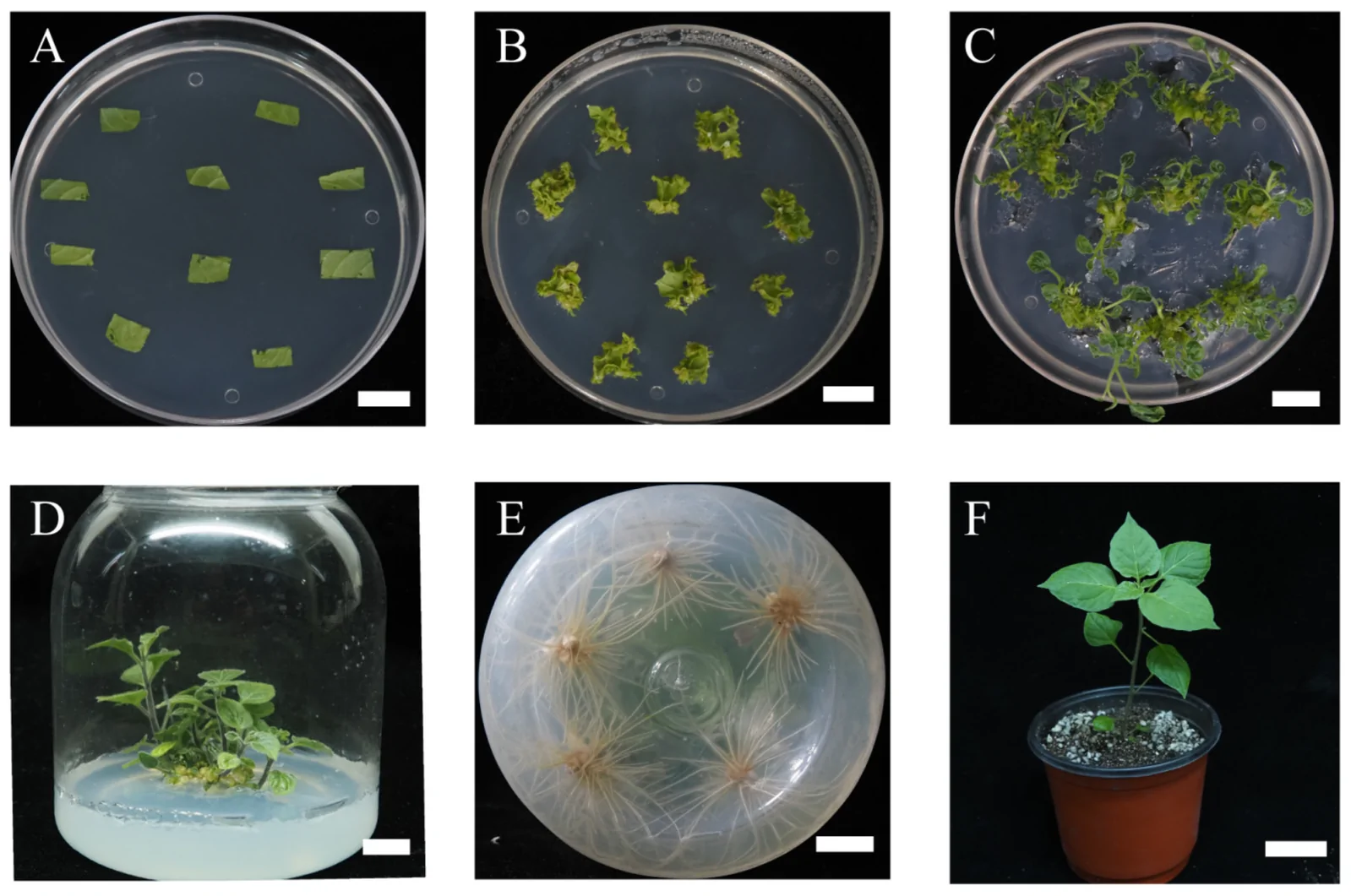
The induction of *Solanum nigrum* L. adventitious shoots via leaves to achieve organogenesis and plant regeneration. (**A**) Leaf explants were inoculated onto MS medium supplemented with 30 g·L^−1^ sucrose, 2.0 mg·L^−1^ 6-BA and 0.1 mg·L^−1^ NAA. Bar = 1 cm. (**B**) On the 12th day, green shoot primordia emerged at the leaf margins, accompanied by a small number of adventitious shoots. Bar = 1 cm. (**C**) The adventitious shoots gradually elongated. Bar = 1 cm. (**D**) The adventitious shoots were inoculated into elongation medium. Bar = 1 cm. (**E**) The rooting of the adventitious shoots. Bar = 1 cm; (**F**) Transplantation of plantlets in sand-soil mixture. Bar = 3 cm.

After 14 days of acclimatization and cultivation, the transplant survival rate of in vitro plantlets was counted uniformly. The survival percentage was calculated as the proportion of surviving seedlings relative to the total number of transplanted plantlets. The final transplant survival rate reached 100.00%. All regenerated plantlets grew vigorously with glossy green leaves and multiple newly emerged leaves (Figure 6).

### 2.8. DNA Ploidy Analysis

**Figure 7:**
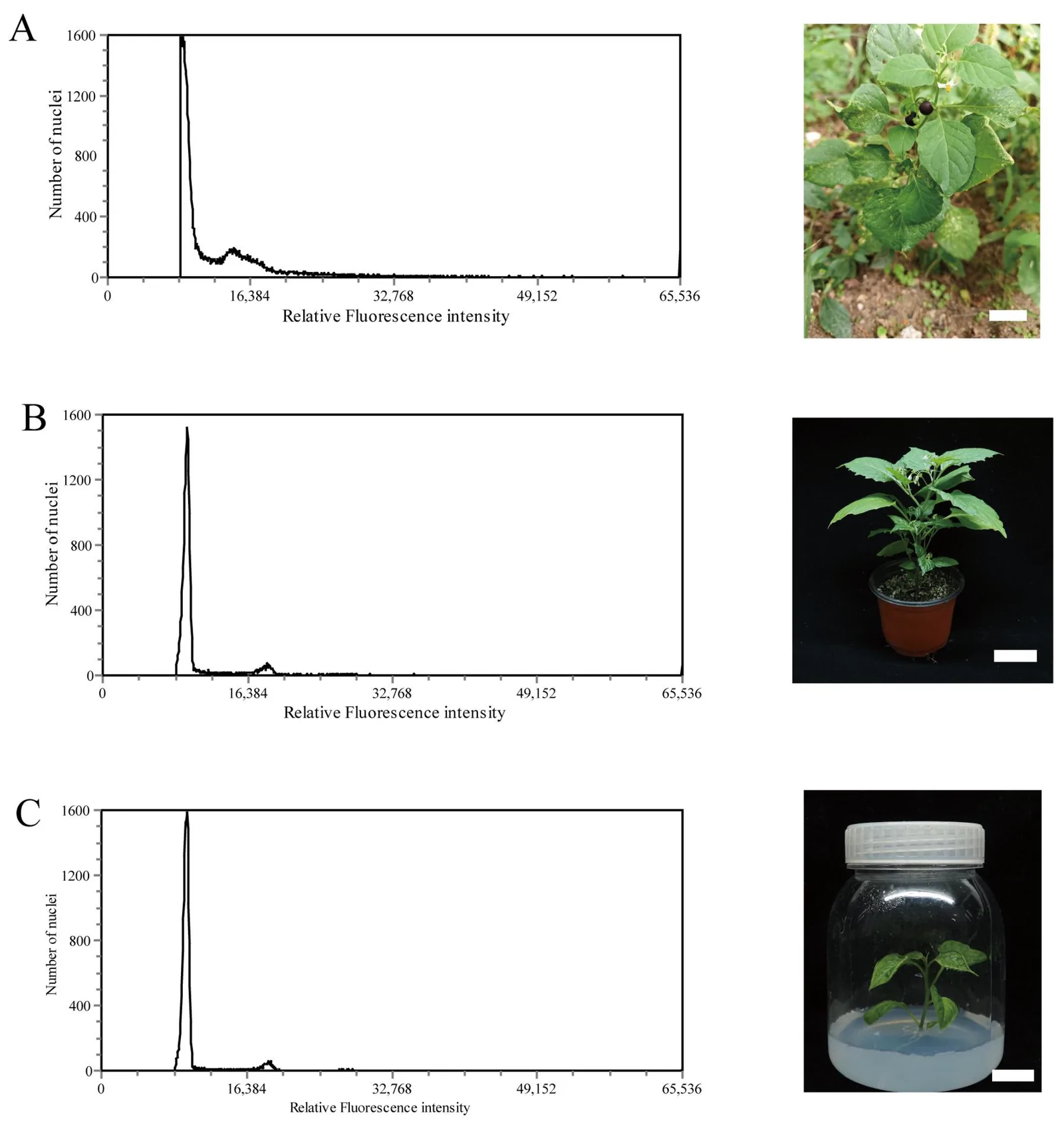
Histograms of relative fluorescence intensity obtained through flow cytometry. (**A**) Wild plants. Bar = 5 cm. (**B**) Plantlets were regenerated from *S. nigrum* seeds. Bar = 2 cm. (**C**) Regenerated plants from adventitious shoots. Bar = 6 cm.

Ploidy levels of wild plants (Figure 7A), seedlings germinated from seeds (Figure 7B), and regenerated *Solanum nigrum* plants (Figure 7C) were determined using a ploidy analyzer. As *S. nigrum* is a hexaploid species [12], fluorescence intensities corresponding to the main DNA peaks of the three groups of materials were identical. Low-amplitude trailing signals on the right side of each histogram represented normal cells at the G2/M phase of the cell cycle and were not indicative of ploidy variation. Adventitious shoots induced with plant growth regulators exhibited no alterations in plant ploidy.

### 2.9. Histological Observations

**Figure 8:**
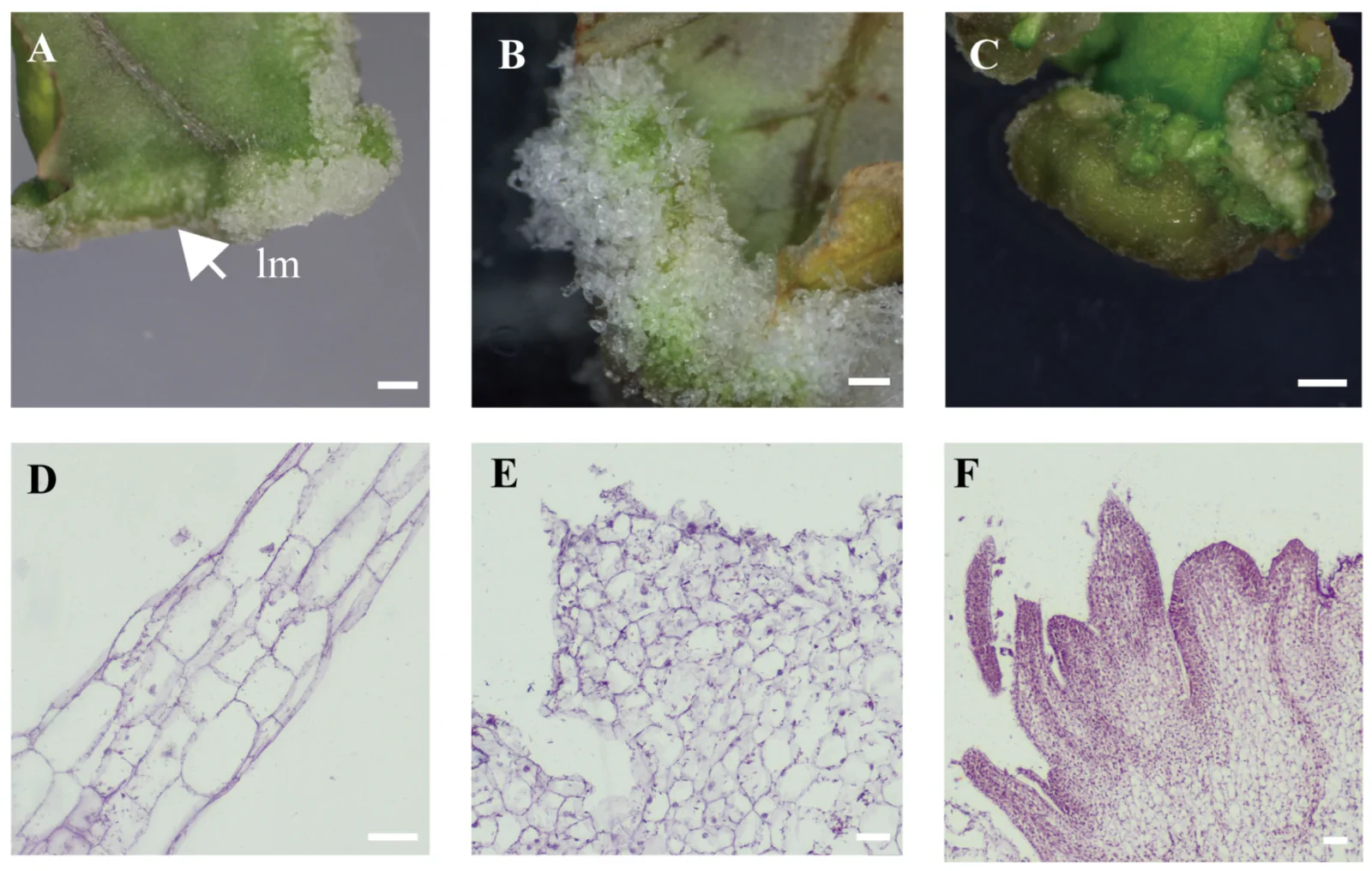
Histological sections of S. nigrum leaf explants stained with hematoxylin. (**A**) Swollen leaf margin with primary loose white calluses. Leaf margin = lm. (**B**) Massive proliferation of calluses at leaf wound sites; partial calluses turned green and became compact. (**C**) Compact green meristematic protrusions (early shoot primordia) differentiated on the surface of calluses, with tender adventitious shoot emerging. Bar = 500 μm. (**D**–**F**): Paraffin histological micrographs of the leaf explants, corresponding to the adventitious shoot developmental stages shown in (**A**–**C**). (**D**) Intact parenchymal cells at the leaf margin at the early culture stage. (**E**) Loose, highly vacuolated callus cells derived from the cell division of leaf subepidermal cells. Bar = 50 μm. (**F**) Densely stained, tightly arranged meristematic cell clusters that developed into intact adventitious shoot primordia. Bar = 200 μm.

Histological analysis characterized the process of adventitious shoot induction in *S. nigrum*. After 8 days of leaf culture on MS medium supplemented with 2.0 mg·L^−1^ 6-BA and 0.1 mg·L^−1^ NAA, swelling emerged at partial leaf margins, accompanied by loose white callus formation (Figure 8A,D). As the culture duration extended, the amount of white calluses gradually increased. Portions of the calluses turned green and became compact, which further differentiated into shoot primordia (Figure 8B,E). After 20 days of cultivation, tender adventitious shoots developed from the green shoot primordia (Figure 8C,F).

## 3. Discussion

The whole plant of *S. nigrum* possesses medicinal value and is rich in various active components, playing important roles in anticancer, anti-inflammatory, and anti-oxidant activities [13,14,15]. Tissue culture technology of *S. nigrum* is of great significance for the medicinal industry. However, systematic studies on its tissue culture remain relatively limited. Therefore, *S. nigrum* was selected as research material to screen multiple influencing factors, aiming to establish an efficient regeneration system and identify the key factors governing its tissue culture, thereby providing technical support for the rapid propagation of medicinal plants.

Owing to the differential sensitivity of different explant types to plant growth regulators, appropriate explant selection can improve the induction frequency of adventitious shoots [16,17]. Leaf, stem, and petiole explants were used for adventitious shoot induction, and leaf explants showed the highest induction rate and the greatest number of adventitious shoots, with significant differences compared to the other explant types. The main reason for this result may be that leaf explants have strong meristematic capacity and moderate sensitivity to plant growth regulators, which is consistent with the growth characteristics of Solanaceae plants. Similarly, studies on *Solanum aculeatissimum* and *Solanum nigrum*, both belonging to the same family, showed that leaf explants outperformed other explants in adventitious shoot induction efficiency, quantity, and quality, consistent with the findings of this study [18]. Compared with the work reported by Subba, cytological evidence supplements the definitive clarification of the initiation pathway of adventitious shoots [18]. Instead of the costly TDZ adopted in their protocol, 6-BA and NAA—PGRs widely utilized in industrial micropropagation with lower prices—were applied, which drastically cuts the raw material cost of plant growth regulators for large-scale seedling production. Moreover, the optimized acclimatization procedure achieves a 100% transplant survival rate of regenerated plantlets, markedly raising the finished seedling yield during commercial mass propagation. Indeed, leaf explants are also commonly used in other families and genera, such as *Actinidia eriantha* ‘White’, *Sinningia Hybrida* ‘Isa’s Murmur’ and *Gynura divaricata* (L.) DC [19,20,21].

The ratio and type of plant growth regulators can significantly influence the time required for adventitious shoot induction, improving induction efficiency [22]. The synergistic use of high concentrations of cytokinin and low concentrations of auxin promotes the formation of calluses and adventitious shoots [23,24], which is consistent with results demonstrating that the combination of BA and NAA effectively induced adventitious shoots from leaf explants. The highest adventitious shoot induction rate and maximum number of adventitious shoots were obtained by using a high concentration of cytokinin combined with a low concentration of auxin (2.0 mg·L^−1^ BA and 0.1 mg·L^−1^ NAA) during adventitious shoot induction. Previous studies have demonstrated that NAA concentrations exceeding 0.1 mg·L^−1^ inhibit shoot formation and root regeneration [25]. Consistently, gradual increases in NAA concentration restrict adventitious shoot formation and root elongation.

Basal culture media vary in nutritional composition and are generally classified into low-salt types (WS, White medium), medium-salt types (Miller medium), and high-salt types (LS, MS, B5 medium) [26]. As a high-salt medium with comprehensive nutritional components, MS medium can satisfy the differentiation requirements of diverse plant species [27,28]. Yaroshko et al. [7] successfully induced calluses and adventitious shoots from different lines of *Solanum lycopersicum* L. using full-strength MS medium. For example, in the study of *Hemerocallis citrina* Baroni, the adventitious shoot induction efficiency on full-strength MS medium was significantly higher than that obtained on half-strength MS medium [29]. Research on *Phyllanthus niruri* also reported that full-strength MS medium achieved optimal performance in terms of adventitious shoot number and shoot length when inducing shoots from stem explants [30]. Therefore, MS medium is widely used for the induction and culture of calluses and adventitious shoots. Sucrose is a commonly used carbon source in plant tissue culture, and its typical concentration is 30 g·L^−1^, which can provide sufficient carbon sources and maintain the osmotic pressure of the medium, facilitating shoot induction [31,32,33,34]. MS medium supplemented with 30 g·L^−1^ sucrose was adopted to induce adventitious shoot formation in *Solanum nigrum* L., and optimal induction effect was achieved.

An efficient regeneration system of *S. nigrum* established via indirect organogenesis exhibits broad application prospects. This system features a high proliferation coefficient and short culture cycle, which enables large-scale mass propagation of high-quality *S. nigrum* seedlings. It effectively overcomes the drawbacks of conventional seed propagation, including uneven seedling emergence and trait segregation, and stably produces clonal plantlets with uniform genetic traits. This provides abundant high-quality seedlings for standardized and normalized artificial cultivation to produce medicinal raw materials of *S. nigrum*.

There are several limitations to the study. For instance, the callus induction cycle is relatively long during indirect adventitious shoot organogenesis. In the rooting stage, high concentrations of NAA tend to trigger abnormal root formation compared with the PGR-free medium. Although the biological replicates adopted were sufficient to support the statistical validity and repeatability of the core findings, the sample size was relatively limited compared with large-scale screening experiments. Further increases in sample quantity and expanded experimental replicates will be conducted in future studies to verify the stability and universality of the regeneration protocol, and to further optimize the culture system of *Solanum nigrum* L.

Somaclonal variation is a common risk in plant tissue culture, particularly under long-term subculture. A short-term organogenesis regeneration system with limited subculture cycles was applied to reduce the accumulation of somaclonal mutations. Obvious somaclonal variation was assessed by morphological observation and flow cytometric ploidy analysis. No detectable phenotypic abnormalities or ploidy shifts were observed in regenerated plantlets. Nevertheless, this approach cannot exclude minor sequence-level mutations. The absence of molecular marker-based genetic fidelity assessment represents a limitation of the present work. Further studies using ISSR or SSR markers will be carried out to comprehensively evaluate the genetic fidelity of regenerants. These practical constraints remain to be further optimized and improved in follow-up research.

## 4. Materials and Methods

### 4.1. Plant Material and Seed Sterilization

Mature seeds of *Solanum nigrum* L. were collected from the botanical garden of the College of Pharmacy, Harbin University of Commerce.

Mature seeds were rinsed under running tap water for approximately 3 h to remove surface mucilage and debris on the seed coat. Subsequently, under aseptic conditions in a laminar flow hood (Beijing Donglianhaer Instrument Manufacturing Co., Ltd., Beijing, China), the seeds were rinsed twice with sterile distilled water, followed by one rinse in 75% (*v*/*v*) ethanol (Lierkang Medical Technology Co., Ltd., Shandong, China) for 5 min each. The seeds were then immersed in 0.1% mercuric chloride (HgCl_2_) (Xilong Scientific Co., Ltd., Shantou, China) solution for 7 min with constant agitation to ensure thorough surface sterilization, and finally rinsed twice with sterile distilled water for 5 min each. The sterilized seeds were evenly spread on sterile filter paper moistened with MS liquid medium ( Beijing Coolaber Science & Technology Co., Ltd., Beijing, China) supplemented with 30 g·L^−1^ sucrose (Sinopharm Chemical Reagent Co., Ltd. Shanghai, China). No plant growth regulators or agar were added to this MS liquid medium. For seed germination, three independent biological replicates were set up, with 100 seeds per replicate. Seedlings were cultured in a growth room at 25 ± 2 °C, with an 18/6 h light/dark photoperiod. The photosynthetic photon flux density (PPFD) across the culture shelf ranged from 180 to 234 μmol·m^−2^·s^−1^, and the corresponding photosynthetically active radiation (PAR) irradiance varied between 39 and 51 W/m^2^. The germinated sterile seedlings were inoculated onto half-strength MS medium for cultivation in subsequent experiments.

### 4.2. Induction of Adventitious Shoots

Stem segments (1 ± 0.1 cm long), leaves (0.5–1.0 cm^2^), and petioles (1 ± 0.1 cm long) were collected from sterile *S. nigrum* seedlings at 30 ± 2 days after sowing (harvested within the time window of 28–32 days after seed sowing) as explants. These explants were inoculated onto MS medium supplemented with 0.7% (*w*/*v*) agar (Xilong Scientific Co., Ltd., Shantou, China), 30 g·L^−1^ sucrose, 2.0 mg·L^−1^ 6-benzyladenine (BA) (Beijing Biotopped Technology Co., Ltd., Beijing, China)and 0.1 mg·L^−1^ α-naphthaleneacetic acid (NAA) (Beijing Biotopped Technology Co., Ltd., Beijing, China) for adventitious shoot induction. The pH of all media was adjusted to 5.8 before autoclaving (Zealway Instrument Inc., Xiamen, China) at 121 °C for 15 min. The cultures were maintained under conditions of 18/6 h (light/dark photoperiod) and a temperature of 25 ± 2 °C. Each treatment included three Petri dishes serving as independent biological replicates, with ten explants cultured per dish, resulting in 30 explants in total per treatment. After 30 days of culture, the adventitious shoot induction rate and the number of adventitious shoots per explant were recorded. The identical experimental setup was used for all subsequent experiments.

Following the explant screening assay, leaf explants were chosen for all subsequent single-factor optimization experiments. Leaves from sterile seedlings of *S. nigrum* were inoculated onto 1/2 MS, MS, White medium (White) ( Beijing Coolaber Science & Technology Co., Ltd., Beijing, China), Gamborg‘s B5 medium (B5) ( Beijing Coolaber Science & Technology Co., Ltd., Beijing, China), and Chu’s N6 medium (N6) ( Beijing Coolaber Science & Technology Co., Ltd., Beijing, China), each supplemented with 0.7% (*w*/*v*) agar, 30 g·L^−1^ sucrose, 2.0 mg·L^−1^ 6-BA and 0.1 mg·L^−1^ NAA. The cultures were maintained under the same conditions as described above. Each treatment included three Petri dishes serving as independent biological replicates, with ten explants cultured per dish, resulting in 30 explants in total per treatment. After 30 days, the adventitious shoot induction rate and shoot number were recorded.

Leaves from sterile seedlings of *S. nigrum* were used as explants and cultured on MS basal medium supplemented with 0.7% (*w*/*v*) agar, 30 g·L^−1^ sucrose and different plant growth regulators (PGRs). A full factorial combination of (0.5, 1.0, 1.5 and 2.0 mg·L^−1^) 6-BA and (0.1, 0.2, 0.5 and 1.0 mg·L^−1^) NAA was tested. The cultures were maintained under the same conditions as described above. Each treatment included three Petri dishes serving as independent biological replicates, with ten explants cultured per dish, resulting in 30 explants in total per treatment. After 30 days, the adventitious shoot induction rate and shoot number were recorded.

To evaluate the effect of sucrose concentration on adventitious shoot induction, leaf explants were cultured on MS basal medium containing 2.0 mg·L^−1^ 6-BA and 0.1 mg·L^−1^ NAA, with sucrose set at 10, 20, 30 and 40 g·L^−1^. The cultures were maintained under the same conditions as described above. Each treatment included three Petri dishes serving as independent biological replicates, with ten explants cultured per dish, resulting in 30 explants in total per treatment. After 30 days, the adventitious shoot induction rate and shoot number were recorded.

### 4.3. Shoot Elongation

The shoot elongation experiment was conducted using adventitious shoots regenerated from leaf explants in the preceding adventitious shoot induction assay. Adventitious shoots with a stem length of approximately 1.0 cm were transferred onto MS basal medium supplemented with 0.7% (*w*/*v*) agar and 0.5 mg·L^−1^ 6-BA combined with four NAA concentrations (0.1, 0.2, 0.4 and 0.5 mg·L^−1^). Each treatment included three independent biological replicates. Each replicate contained ten culture bottles with one explant inoculated per bottle. Cultures were maintained under the same conditions as described above. After 20 days, the number of adventitious shoots and their growth status were recorded.

### 4.4. Rooting

The rooting experiment was conducted using elongated adventitious shoots obtained from the preceding shoot elongation assay. Elongated adventitious shoots were excised and inoculated onto 1/2 MS medium supplemented with 0.7% (*w*/*v*) agar, 30 g·L^−1^ sucrose and four different concentrations of NAA (0.1, 0.5, 1.0 and 1.5 mg·L^−1^). Each treatment included three culture bottles serving as independent biological replicates, with five explants inoculated per bottle. The cultures were maintained under the same conditions as described above. After 20 days, the rooting status was observed, and the rooting rate as well as the average number of roots per shoot were recorded.

### 4.5. Transplantation

Intact regenerated plantlets with well-developed root systems and approximately 15 cm height were kept in sealed culture bottles for 3 days. Subsequently, these seedlings were transplanted into an autoclaved sand–soil mixture (sand: garden soil = 1:3, *v*/*v*), which had been sterilized by autoclaving at 121 °C for 20 min. Plantlets were cultivated in the soil culture room of the College of Forestry, Northeast Forestry University. The illumination system adopted NVC YZ28-T5 28 W 6500 K triphosphor T5 fluorescent tubes manufactured by Huizhou NVC Optoelectronics Technology Co., Ltd. (Huizhou, China) Photosynthetic photon flux density (PPFD) was measured at multiple points at the canopy height of *S. nigrum* plants. The average PPFD during the entire cultivation period ranged from 180 to 234 μmol·m^−2^·s^−1^. The photoperiod was set as 16 h light/8 h dark, the cultivation temperature was maintained at (25 ± 2) °C, and the relative air humidity was kept at 35%. The substrate was sprayed with clean water every 3 days to maintain moderate moisture and prevent root rot caused by waterlogging.

### 4.6. DNA Ploidy Analysis

To determine whether the induced adventitious shoots had undergone chromosomal variation, a ploidy analysis of the regenerated seedlings was performed using a ploidy analyzer (CyFlow Cube 6, Sysmex Partec GmbH, Görlitz, Germany). Ten plantlets were randomly sampled from regenerated seedlings, seed-grown plants and wild plants respectively for ploidy analysis. Fresh leaves to be tested were excised with a blade and placed in a Petri dish. Then, 200 μL of staining solution was added dropwise to the leaf surface, and the tissue was chopped thoroughly with the blade. After lysis and extraction for 1 min, an additional 1200 μL of staining solution was added for staining for 1 min. Finally, the suspension was filtered to prepare the sample solution, which was subsequently subjected to ploidy analysis using the ploidy analyzer. The hexaploid wild-type *S. nigrum* was used as the reference control rather than an exogenous internal standard. Wild-type samples were assayed in each batch of flow cytometry runs to define the standard fluorescence position for hexaploid G0/G1 nuclei. Regenerated samples were compared against this reference. Samples with a relative fluorescence deviation of less than 5% relative to the reference peak were scored as hexaploid. The coefficient of variation (*CV*) of each nuclear peak was controlled below 5% to guarantee reliable peak shape. Samples with overlapping peaks, excessive debris interference or *CV* values exceeding 5% were eliminated during data filtering.

### 4.7. Histological Analysis

Leaf explants at three successive developmental stages were collected, including initial leaf margin swelling, callus formation at leaf margin, and shoot primordium initiation from calluses. These explants were immersed in FAA fixative solution. After thorough degassing, samples were fixed at 4 °C for 24 h for subsequent histological analysis. The fixed samples were subjected to serial ethanol dehydration with 50%, 70%, 80%, 90% and absolute ethanol for 1.5 h at each concentration. After dehydration, the samples were cleared sequentially in an equal-volume mixture of xylene and absolute ethanol (volume ratio = 1:1) and pure xylene for 1 h each. The cleared tissues were embedded in paraffin wax at 65 to 70 °C. Tissue sections were prepared using a microtome (HM 340 E, Microm GmbH, Waldorf, Germany). Sections were mounted on glass slides and baked in an oven at 45 °C for 3 days. Paraffin sections were dewaxed via a serial gradient of xylene, absolute ethanol, 95% ethanol, 80% ethanol and 75% ethanol. The dewaxed sections were stained with 0.5% hematoxylinsolution for 3 min, then observed and photographed under an optical microscope (Docuval, Carl Zeiss, Oberkochen, Germany). For each developmental stage, three independent biological replicates were included, and two sections were prepared per replicate (six sections in total). To avoid selection bias during image acquisition, micrographs were randomly captured from non-overlapping regions on each section instead of deliberately selecting fields with optimal morphology.

### 4.8. Statistical Analyses

Adventitious shoot induction rate (%) = (Number of explants producing adventitious shoots/Total number of inoculated explants) × 100%

Average number of elongated shoots = Total number of shoots longer than 2.0 cm/Total number of adventitious shoots at inoculation

Average number of adventitious roots per explant = Total number of adventitious roots/Number of rooted explants

Rooting rate (%) = (Number of rooted adventitious shoots/Total number of inoculated adventitious shoots) × 100%

Data processing and statistical analyses were performed using SPSS Statistics 19 and Excel 2021, and all figures were plotted with GraphPad Prism 10.2. Comparisons among multiple groups were performed using one-way analysis of variance (*ANOVA*), followed by post hoc multiple comparisons using Tukey’s test. A *p*-value < 0.05 was considered statistically significant.

## 5. Conclusions

The results showed that leaf explants yielded a significantly higher adventitious shoot induction rate (100.00%) compared with stem segments and petioles, confirming leaf tissue as the optimal explant for *S. nigrum* regeneration system establishment. Using leaves as explants, the optimal medium for adventitious shoot induction was MS medium supplemented with 0.7% (*w*/*v*) agar, 30 g·L^−1^ sucrose, 2.0 mg·L^−1^ 6-BA and 0.1 mg·L^−1^ NAA; the optimal medium for adventitious shoot proliferation was MS medium supplemented with 0.7% (*w*/*v*) agar, 30 g·L^−1^ sucrose, 0.5 mg·L^−1^ 6-BA and 0.1 mg·L^−1^ NAA; and the optimal medium for adventitious shoot elongation was MS medium supplemented with 0.5 mg·L^−1^ 6-BA, 0.1 mg·L^−1^ NAA, 30 g·L^−1^ sucrose and 0.7% (*w*/*v*) agar, which produced an average of 27.67 elongated adventitious shoots per explant. The optimal rooting medium was half-strength MS medium containing 0.1 mg·L^−1^ NAA, 30 g·L^−1^ sucrose and 0.7% (*w*/*v*) agar, with a rooting rate of 96.67%. This protocol significantly improves adventitious shoot induction efficiency and shortens the culture cycle for *S. nigrum*. The established tissue culture system provides a foundation for the rapid mass propagation of this medicinal species and related plants within the genus.

## Figures and Tables

**Table 1:** Effect of explants on adventitious shoot induction.

Explant Type	Average Number of Adventitious Shoots	Shoot Induction (%)
Leaves	9.87 ± 2.00 ^a^	100.00 ± 0.00 ^a^
Petioles	2.20 ± 1.22 ^b^	71.33 ± 5.13 ^b^
Stem segments	2.13 ± 1.63 ^b^	51.67 ± 7.23 ^b^

Note: All data in tables are presented as mean ± standard deviation (SD). Different lowercase letters within the same column indicate significant differences among treatments at *p* < 0.05.

**Table 2:** Effect of basal media on adventitious shoot induction.

Medium Type	Average Number of Adventitious Shoots	Shoot Induction (%)
1/2 MS	5.70 ± 0.26 ^b^	89.33 ± 1.53 ^b^
MS	8.40 ± 0.44 ^a^	100.00 ± 0.00 ^a^
B5	5.00 ± 0.26 ^c^	87.67 ± 3.22 ^bc^
N6	2.30 ± 1.91 ^cd^	76.67 ± 2.08 ^c^
White	0.00 ± 0.00 ^d^	0.00 ± 0.00 ^d^

Note: All data in tables are presented as mean ± standard deviation (SD). Different lowercase letters within the same column indicate significant differences among treatments at *p* < 0.05.

**Table 3:** The two-way ANOVA results for the average number of adventitious shoots affected by BA and NAA.

Variance Source	df	F	*p*
BA	3	180.281	*p* < 0.001
NAA	3	88.433	
BA × NAA	9	14.489	

Levene’s test verified homogeneity of variances (*F* = 1.675, *df*_1_ = 15, *df*_2_ = 32, *p* = 0.108), satisfying the prerequisite for two-way ANOVA.

**Table 4:** Effect of plant growth regulators (PGRs) on adventitious shoot induction.

6-BA (mg·L^−1^)	NAA (mg·L^−1^)	Average Number of Adventitious Shoots	Shoot Induction (%)
0.5	0.1	2.80 ± 0.62 ^de^	16.67 ± 11.55 ^c^
	0.2	2.00 ± 0.70 ^de^	40.00 ± 10.00 ^bc^
	0.5	1.57 ± 0.21 ^e^	70.00 ± 10.00 ^ab^
	1.0	0.83 ± 0.25 ^e^	20.00 ± 10.00 ^c^
1	0.1	5.43 ± 0.15 ^c^	43.33 ± 15.28 ^bc^
	0.2	3.36 ± 0.40 ^d^	66.67 ± 11.55 ^b^
	0.5	3.00 ± 0.62 ^d^	56.67 ± 15.28 ^b^
	1.0	2.20 ± 0.44 ^de^	83.33 ± 11.55 ^ab^
1.5	0.1	5.47 ± 0.51 ^c^	50.00 ± 10.00 ^bc^
	0.2	6.93 ± 0.59 ^bc^	86.67 ± 5.77 ^ab^
	0.5	5.10 ± 0.40 ^c^	63.33 ± 15.28 ^b^
	1.0	4.60 ± 0.20 ^cd^	83.33 ± 5.77 ^ab^
2	0.1	8.97 ± 0.85 ^a^	100.00 ± 0.00 ^a^
	0.2	7.40 ± 0.60 ^b^	93.33 ± 5.77 ^ab^
	0.5	4.43 ± 0.21 ^cd^	91.67 ± 7.64 ^ab^
	1.0	3.07 ± 0.51 ^d^	87.67 ± 13.65 ^ab^

Note: All data in tables are presented as mean ± standard deviation (SD). Different lowercase letters within the same column indicate significant differences among treatments at *p* < 0.05.

**Table 5:** Effect of sucrose on adventitious shoot induction.

Sucrose Concentrations (g·L^−1^)	Average Number of Adventitious Shoots	Shoot Induction (%)
10	4.93 ± 1.61 ^b^	61.33 ± 10.59 ^b^
20	5.43 ± 0.47 ^b^	83.33 ± 5.77 ^ab^
30	8.87 ± 0.38 ^a^	100.00 ± 0.00 ^a^
40	5.50 ± 1.11 ^b^	76.67 ± 5.77 ^b^

Note: All data in tables are presented as mean ± standard deviation (SD). Different lowercase letters within the same column indicate significant differences among treatments at *p* < 0.05.

**Table 6:** Elongation of adventitious shoots.

6-BA (mg·L^−1^)	NAA (mg·L^−1^)	Number of Elongated Single Nodes Per Adventitious Shoot
0.5	0.1	27.67 ± 4.04 ^a^
	0.2	14.33 ± 3.05 ^b^
	0.4	12.00 ± 4.58 ^b^
	0.5	9.67 ± 1.53 ^b^

Note: All data in tables are presented as mean ± standard deviation (SD). Different lowercase letters within the same column indicate significant differences among treatments at *p* < 0.05.

## Data Availability

The original contributions presented in this study are included in the article. Further inquiries can be directed to the corresponding author.

## Abbreviations

The following abbreviations are used in this manuscript:

B5	Gamborg‘s B5 medium
6-BA	6-benzyladenine
CV	Coefficient of variation
FAA	Formalin-Acetic-Acid-Alcohol
MS	Murashige and Skoog
NAA	α-naphthaleneacetic acid
N6	Chu’s N6 medium
PAR	Photosynthetically active radiation
PGRs	Plant growth regulators
PPFD	Photosynthetic photon flux density
TDZ	Thidiazuron
White	White medium
